# Network as transconcept: elements for a conceptual demarcation in the field of public health

**DOI:** 10.1590/S1518-8787.2016050006306

**Published:** 2016-08-16

**Authors:** Carlos Eduardo Menezes Amaral, Maria Lúcia Magalhães Bosi

**Affiliations:** I Programa de Pós-Graduação em Saúde Coletiva. Faculdade de Medicina. Universidade Federal do Ceará. Fortaleza, CE, Brasil; II Departamento de Saúde Comunitária. Faculdade de Medicina. Universidade Federal do Ceará. Fortaleza, CE, Brasil

**Keywords:** Health Care Networks, Health Management, Health Planning, Program Evaluation, Public Health

## Abstract

The main proposal to set up an articulated mode of operation of health services has been the concept of network, which has been appropriated in different ways in the field of public health, as it is used in other disciplinary fields or even taking it from common sense. Amid the diversity of uses and concepts, we recognize the need for rigorous conceptual demarcation about networks in the field of health. Such concern aims to preserve the strategic potential of this concept in the research and planning in the field, overcoming uncertainties and distortions still observed in its discourse-analytic circulation in public health. To this end, we will introduce the current uses of network in different disciplinary fields, emphasizing dialogues with the field of public health. With this, we intend to stimulate discussions about the development of empirical dimensions and analytical models that may allow us to understand the processes produced within and around health networks.

## RESUMO

A principal proposta para configurar um modo articulado de funcionamento dos serviços de saúde tem sido o conceito de rede, que vem sendo apropriado de diferentes formas no campo da saúde coletiva, conforme seu emprego em outros campos disciplinares ou mesmo tomando-o do senso comum. Em meio à pluralidade de usos e concepções, reconhecemos a necessidade de rigorosa demarcação conceitual acerca de redes no campo da saúde. Tal preocupação visa a preservar o potencial estratégico desse conceito na investigação e planificação no campo, superando precariedades e distorções ainda observadas em sua circulação discursivo-analítica na saúde coletiva. Para tanto, apresentaremos os usos correntes de rede em diferentes campos disciplinares, destacando interlocuções com o campo da saúde coletiva. Com isso, pretendemos estimular o debate acerca do desenvolvimento de dimensões empíricas e modelos de análise que permitam compreender os processos produzidos no interior e ao redor das redes de saúde.

## INTRODUCTION

The notion of network as guiding principle of the organization of actions and services of the health sector has been present in Brazil since the ideals of creation of the Brazilian Unified Health System (SUS). We can recognize it in the influence that SUS has received, in its conception, from the British healthcare system and, consequently, from the Dawson report^13^, which introduces the principle of health networks and anticipates the idea of articulated operation between services of different complexities. However, the formal concern with the organization of Healthcare Networks (HCN)^18^ was adopted by the Ministry of Health only in 2010, with Ordinance 4,279, which established guidelines for the organization of HCN. Nevertheless, between these two essential moments – creation of SUS and regulation of HCN –, we observe an intermittent arising of the concept of network under different justifications, aiming different effects with the health system.

The main proposal to set up an articulated mode of operation of health services has been the concept of network. Currently, several speeches use this concept, but without clearly disclosing to what they refer to. This concept has been appropriated in different ways in the field of public health, according to its use in other fields of knowledge or even taking it from common sense. Amid such diversity of uses and conceptions, we recognize the need for rigorous conceptual demarcation about networks in the field of health, by the analysis of the influences on the understandings and uses of the term.

The concept of network occupies a position similar to other strategic concepts to SUS, as comprehensiveness^19^, humanization^2^, quality^22^, or innovation^3^. Such terms present conceptual challenges, requiring considerable theoretical-critical effort to embrace them in their breadth and multidimensionality and, thus, to make them strategic tools for the development of this system. To understand the implications of the networks in the qualification of healthcare, we need the theoretical consolidation of this concept, establishing its limits and its radicalism. By this process, we will be able to recognize empirical evidence of its practical effects, as well as analyze approaches and distances between the conceptual ideality and the materiality of networks in the routine of services and programs that make up the system.

Here, we present the uses of network in different disciplinary fields – whether in a conceptual, analytical, or metaphorical way – and we focus in some dialogues with the field of public health.

## THE MULTIPLE USAGES AND MEANINGS OF NETWORK

The idea of network as a principle that organizes, explains, constitutes, or analyzes phenomena is present in different fields of modern science, such as social sciences, information sciences, philosophy, management, and, more recently, in the health sciences. We can consider, therefore, the network as a transconcept, given its multiple definitions, sometimes divergent, with importance and relative effects of truth in several fields^4^.

The original meaning of the word network, according to a dictionary of the Portuguese language^15^ (HOUAISS, 2001, p. 2,406), is “intertwined of threads (...), cords, wires, etc., forming a kind of open mesh fabric, composed in diamonds or squares of different sizes”. According to the dictionary, other meanings derive from this concrete object, such as: “set of points that communicate with each other”; “set of people or establishments that maintain contact with each other, usually organized and under a single command” or “interweaving of structures such as blood vessels, muscle fibers, nerves etc.”.

The definitions show as their semantic core the idea that something is created by communication, contact, interweaving, or other forms of relation between elements, establishing new possibilities based on the structure thus produced. This driving idea allows the application of the notion of network in various situations, as the continuation of the entry in the dictionary points out, when referencing aspects of connection, articulation, association, communication, interdependence, and group.

In the scientific scope, the initial application of the concept of network belongs to the social sciences, with Georg Simmel (1858-1918) using the term to explain the emerging way of life in metropolises. The beginning of the systematic use of the term is identified in the sociometry of Jacob Moreno, in the 1930s, in studies and interventions in small groups, and in the writings of Radcliffe-Brown, from the same period, describing the social structure as a network of relationships institutionally determined^1^ ^,^ ^23^.

The network as an analytical perspective was developed in the field of anthropology by John Barnes, in his criticism to the use of isolated characteristics of individuals or institutions seeking to explain life in society. Thus, he advocates network as a fundamental analytical category, identifying its size, number of elements, degree of density and symmetry of relations^1^. This approach has developed vigorously in anthropology and sociology, incorporating ethnographic references and mathematical modelings in the studies of relationships, giving rise to the social network analysis. The conceptual accuracy of this perspective allows stating that the social network analysis is not merely a vocabulary of intuitive or metaphorical appeal, but a precise way of defining social concepts and a theoretical framework for testing theories on social relationships^23^.

Milton Santos^20^, in analyzing the globalized society, also uses the concept of network to explain a new constitution of the social space-time. He argues that the network has a material aspect, consisting in the infrastructure for the transport of matter, information, or energy, and a social and political aspect, composed of the values, messages, and people attending the network. While analytical dimensions, he proposes a “genetic approach”, identifying the history of additions and removals of elements in the network and a “current approach”, describing its constituents elements, its quantities and qualities, and its relations with social life.

Manuel Castells highlights the logic of networks as a key aspect of a new sociotechnical paradigm: the paradigm of information. He advocates that the revolution in information technology is “at least, a historic event with the same importance as the industrial revolution of the 18th century, inducing a pattern of discontinuity in the material bases of economy, society, and culture” (p. 50)^5^. Thus, the modes of organization of the human production and sociability are determined by a logic made possible by the development of informational technologies, and the morphology of network is necessary to the growing complexity of interaction, allowing to “structure the unstructured, preserving the flexibility” (p. 78)^5^.

In the field of information sciences, the topic of networks is quite present: since the beginning of computing, with the communication of great mainframes to smaller terminals that in the future would create the independent personal computers, until the current ubiquity of connection between computers, by the internet, turning the expression “social networks” another current use of the term^9^.

Besides the material and functional aspect, the great potential of networks is their almost unlimited capacity of connections between different points. According to Castells^5^, networks are open structures with possibility of unlimited expansion, as long as the new nodes share common communication codes. This notion of endless connections is also present in the philosophy of Deleuze and Guattari^10^, with the notion of “rhizome” while open system of concepts. In this logic, the concepts would be related to circumstances, and not to essences, allowing permanent invention and re-creation^11^. The rhizome radicalizes the heterogeneity of the network, challenging the need for common codes as the basis for its connection, allowing the reinvention of its own conditions of engagement by contextual, and not aprioristic, features.

An important question inherent to the concept of network would be, then, its conditions of determination. Even if the affirmation of multiplicity^10^, plurality^5^, and deregulation^20^ present in the networks is genuine, this does not mean absence of determination. It implies, instead, the multiplication of normativeness, in which every local rule has interferences from rules from other places.

It is possible to recognize that the notion of network, regardless of the disciplinary distances between the presented authors, has some common parameters, mainly about the production of effects by its connection possibilities. However, networks can be considered in terms of the regularity and recurrence of such connections, or of the heterogeneity, unpredictability, and generative capacity of entanglements. We can still state the network as a possibility for sharing material and informational resources, by the standardization of codes and rules, or as the space of dissemination of the new and non-hegemonic, restructuring its architecture according to the uniqueness of its contents.

## NETWORK: INTERFACES WITH PUBLIC HEALTH

Before this multiplicity of characteristics of the term network, we inquire: how can this transconcept be incorporated into the field of public health without losing its wideness and complexity? To propose a demarcation that dialogues to the public health and its objects, we performed an unsystematic review of the literature, recovering 16 studies of the field presenting concepts or dimensions of network. The unsystematic mode was necessary, because the definition of the concept of network is so incipient that the articles use different descriptors: health network, healthcare networks, services network etc. Besides, an important part of the literature is in books and chapters that do not use descriptors.

Presenting this review in detail is beyond scope of this text, but we intend to present a composition comprising the dimensions found in the field of public health and in other fields, from the contrast and overlap of different definitions found. Thus, we came to the concept of network as a state of relationship between elements, produced and organized by a central rationality or independent of it, always with different normative vectors. Therefore, the network can be constituted from a declared and aprioristic design, which causes and organizes an approximation between elements^17^, but simultaneously adds partial, convergent, or antagonistic intentionalities^12^, and may dispense with a central goal. The studies that approach networks advocating the coordination for primary healthcare, regionalization, and integration^6^ ^,^ ^16^ ^,^ ^21^ highlight the rational and pragmatic character of networks and their capacity for planning and ordering. Other studies, in considering the services networks as one more device of the networks of sociability and subjectivity of users and professionals^7^ ^,^ ^24^, highlight its opposite aspect, of rupture and production of heterogeneity, so that more sociological approaches of network analysis, in the form of social networks, would emerge as analytical possibility^1^ ^,^ ^23^.

This apparent paradox arises from the nature of each network element that, as well as its interrelationships, are extremely varied, demanding to be understood in their uniqueness. The same applies to the effects arising from each individual relationship and from the set of these relationships^18^. We emphasize that these effects can focus both on the network interiority – in its elements or relationships – and in its exteriority, even if marking the boundaries of a network is always an artificial construction, carried out according to the intentionality of the analysis, as already highlighted by authors from other fields^10^ ^,^ ^20^.

To investigate or evaluate health networks, it is necessary to consider the multiple constituent aspects of this virtual space^5^ ^,^ ^20^, bringing the need for models that consider the properties of the minimum elements of the network in question: Which and how many services make up the network? Which is the proposal of care and level of complexity of each service? What is its territory of action and liability limit? How does the access work? However, the model must simultaneously incorporate what is established in the connections between services (referrals, support modalities, communication), describing the conditions and effects of each type of relationship.

The set of these individual and relational attributes produce effects on the network integration (cooperation, complementarity, competition), requiring an appropriate evaluation that cannot be reduced to the mere juxtaposition of isolated assessments of the various services. It is necessary to investigate the occurrence of a common governance among autonomous services, which makes possible its cooperation in a collective clinical project^14^, and the occurrence of lack of harmony, which highlight the disjunction of the temporality and expectations of managers, professionals, and users^8^.

The research references need still to include the normativeness of the network, which refers both to the production and use of clinical guidelines and formal care lines and to the production of informal criteria of inclusion and exclusion in each point of the network, which together will determine the transit of people, information, and inputs. The diverse rationalities in the networks are also constituted by the circulation of values and meanings, shared or not by their managers, operators, users, and non-users, which cross and modify the other aspects of the network.

A conceptual standpoint to networks, which is the goal of this study, does not aim to simply show a correspondence of the literature with current health networks characteristics, or even with the models idealized in the recent planning proposals. The conceptual demarcation aims at a generalization capability that allows both to recognize and anticipate limits and potentials of the (real) instrumental operationalizations of the networks. With this, our objective is to stimulate the discussion for the development of empirical dimensions of analysis of networks that are not reduced to assess their suitability to guidelines and indicators of coverage, but that allow to understand the processes generated within and around it. We intend to contribute to a systematic and consequent reflection, aimed at the preservation of the strategic potential of this concept in the analyses and planning in the field, overcoming uncertainties and distortions still observed in its discursive-analytical circulation in the public health.
